# Hereditary angioedema: beyond international consensus - circa December 2010 - The Canadian Society of Allergy and Clinical Immunology Dr. David McCourtie Lecture

**DOI:** 10.1186/1710-1492-7-1

**Published:** 2011-02-10

**Authors:** Tom Bowen

**Affiliations:** 1 Clinical Professor of Medicine and Paediatrics, University of Calgary, 705 South Tower 3031 Hospital Dr. NW, Calgary, Alberta, T2N 2T8, Canada

## Abstract

**Background:**

The 2010 International Consensus Algorithm for the Diagnosis, Therapy and Management of Hereditary Angioedema was published earlier this year in this Journal (Bowen et al. *Allergy, Asthma & Clinical Immunology *2010, 6:24 - http://www.aacijournal.com/content/6/1/24). Since that publication, there have been multiple phase III clinical trials published on either prophylaxis or therapy of hereditary angioedema and some of these products have changed approval status in various countries. This manuscript was prepared to review and update the management of hereditary angioedema.

**Objective:**

To review approaches for the diagnosis and management of hereditary angioedema (HAE) circa December 2010 and present thoughts on moving from HAE management from international evidence-based consensus to facilitate more local health unit considerations balancing costs, efficacies of treatments, and risk benefits. Thoughts will reflect Canadian and international experiences.

**Methods:**

PubMed searches including hereditary angioedema and diagnosis, therapy, management and consensus were reviewed as well as press releases from various pharmaceutical companies to early December 2010.

**Results:**

The 2010 International Consensus Algorithms for the Diagnosis, Therapy and Management of Hereditary Angioedema is reviewed in light of the newly published phase III Clinical trials for prevention and therapy of HAE. Management approaches and models are discussed.

**Conclusions:**

Consensus approach and double-blind placebo controlled trials are only interim guides to a complex disorder such as HAE and should be replaced as soon as possible with large phase IV clinical trials, meta analyses, data base registry validation of approaches including quality of life and cost benefit analyses, safety, and head-to-head clinical trials investigating superiority or non-inferiority comparisons of available approaches. Since not all therapeutic products are available in all jurisdictions and since health care delivery approaches and philosophy vary between countries, each health care delivery sector will likely devise their own algorithms based on local practicalities for implementing evidence-based guidelines and standards for HAE disease management. Quality-of-life and cost affordability benefit conclusions will likely vary between countries and health care units. Data base registries for rare disorders like HAE should be used to detect early adverse events for new therapies and to facilitate phase IV clinical trials and encourage superiority and non-inferiority comparisons of HAE management approaches.

## Introduction

The 2010 International Consensus Algorithm for the Diagnosis, Therapy and Management of Hereditary Angioedema was published earlier this year in this Journal [1].. Since that publication, there have been multiple phase III clinical trials and other studies published on either prophylaxis or therapy of hereditary angioedema and some of these products have changed licensure status in various countries. With publication of these clinical trial results [2-8], Dr. Marco Cicardi convened an evidence-based consensus meeting in Italy, September 2010 and his group is preparing manuscript(s) for publication of those proceedings. This manuscript will explore some other disease management models and experiences and reflect on application of some of this experience to management of HAE particularly in Canada and will propose updates to the 2010 Consensus algorithms circa December 2010.

The clinical characteristics and management of hereditary angioedema (HAE) due to C1 inhibitor deficiency (HAE-C1INH) including diagnosis, swelling event prophylaxis, and swelling event therapy has been reviewed in many previous publications including the three international consensus documents [1]. HAE-C1INH patients lack C1INH functional activity and may develop recurrent nonpruritic swelling of skin and submucosal tissues eliciting associated pain syndromes, nausea, vomiting, diarrhea, and life-threatening airway swellings. Untreated airway angioedema has an associated significant risk of dying from asphyxia. The first angioedema may be a life-threatening airway edema event. Although prodromal serpiginous erythematous rashing is sometimes seen, pruritic urticaria usually makes the diagnosis of HAE unlikely. The HAE-C1INH gene maps to chromosome 11q12-q13.1 with autosomal dominant genetics and 25% spontaneous mutation and little or no genotype-phenotype correlation. The genetic protein defect was described by Donaldson in 1963. Acquired angioedema forms described in 1972 and differs from HAE having absent family history, late onset of symptoms, usually low C1q antigen levels and includes drug-induced angioedema (e.g. angiotensin-converting enzyme inhibitors, ACE-I) are not the focus of this article. The incidence of HAE is approximately 1:50,000 with no ethnic group differences. Two forms of HAE-C1INH have been described: type I HAE with low C1INH antigenic protein and functional activity (85% of cases) and type II HAE with normal or elevated protein but low C1INH function (15% of cases). Another less common type of HAE expresses normal C1-INH (sometimes referred to as type III HAE) with the defects yet to be identified. The pathophysiology of HAE-C1INH types I and II appears to relate to bradykinin resulting in angioedema. HAE may present under one year of age with laryngeal attacks uncommon before age three and tending to occur later than other symptoms. Angioedema events often worsen with Untreated attacks typically last over 48 to 96 hours. Attack triggers may include puberty, estrogen-containing contraceptives, hormone replacement therapy, menstruation, pregnancy, stress, infections, ACE-inhibitors, minor trauma, but triggers are often unidentified with attacks varying from periodic, clustering, and variable periods of remission. Angioedema attacks do not respond to treatment with glucocorticoids or antihistamines, and epinephrine has only at best a transient and minimal benefit.

When the first HAE consensus meeting took place in Toronto, Canada in October 2003, there were no licensed drugs in North America for the treatment of HAE attacks and only two randomized clinical trials with plasma-derived C1 inhibitor replacement therapy (pdC1INH;[9,10]) and a few clinical trials using androgens and antifibrinolytics [11-13]. C1-esterase inhibitor concentrates (Berinert P^® ^and Cetor^®^) were available mostly in Europe at the time. There have been two subsequent international consensus documents published including the 2010 International Consensus Algorithm for the Diagnosis, Therapy and Management of Hereditary Angioedema [1]. Since that publication there are now several phase III clinical trials recently published in HAE prophylaxis and therapy and these have led to the licensing of pdC1INH (Berinert^®^, CSL Behring; Cinryze^®^, ViroPharma; Cetor-n^®^, Sanquin) in many parts of the world; bradykinin receptor antagonist (Icatibant, Firazyr^®^, Jerini/Shire) in Europe; kallikrein inhibitor (Ecallantide, Kalbitor^®^, Dyax) in the United States; and recombinant C1-INH replacement therapy (rhC1INH; conestat alfa; Rhucin^®^, Pharming) in Europe [2-8]. Tranexamic acid has been showed to be relatively ineffective therapy [14]. Danazol prophylaxis remains an option but therapeutic agents are now being used more for prophylaxis because of danazol adverse events [15-17]. With the results of these phase III trials, Dr. Cicardi's group is preparing the evidence-based consensus. This manuscript is meant to reflect on other disease management models, apply some of these thoughts to HAE management as it might apply to a local health unit model such as the Canadian Health Care System and update the algorithms from the 2010 International Consensus document given the new clinical trial data circa December 2010.

### HAE Management: Learning from other disease management models

HAE management approaches can draw on the experiences of other disease management approaches. With current modern therapies, approaches to diseases like HAE, hemophilia, and immunedeficiency now aim to normalize lives of such patients and not merely treat acute events such as swelling, bleeding, or infection.

#### Hemophilia Model

Similar to hemophilia A and B, HAE results from a deficiency in a plasma protein that interacts with many homeostasis pathways in the human body (C1INH interacts with the complement, coagulation, contact and fibrinolysis pathways). The incidence of HAE is similar to hemophilia A and B. Similar to hemophilia, one of the early approaches to HAE angioedema events is replacing the missing plasma protein with concentrates made from human blood donations. Initially single donor plasma products were utilized and these were replaced by multiple donor concentrates. These concentrates suffered from increased risk of transfusion transmitted viral events similar to the hemophilia replacement concentrates. With the introduction of pasteurization of Berinert^® ^C1INH replacement therapy (Berinert P^®^) in 1985, viral transmission was contained similar to hemophilia product improvements. Currently available pdC1INH products now have a lengthy well documented and impressive safety record. As with hemophilia and partially because of the fear of transmitting various blood borne pathogens, IV recombinant replacement products have been developed (rhC1INH). Starting in 1973, home care for hemophilia directed through comprehensive care clinics was developed and home self or assisted infusion of concentrates evolved. However, home care and self-infusion programs have been slow to develop in HAE for no apparent reason except physicians caring for HAE patients were usually allergists immunologists or other specialists not experienced in the hemophilia model. The quickest approach to developing HAE home care and self or assisted infusion models is to emulate the hemophilia comprehensive treatment and home care models. They have years of experience in education for self and assisted infusion programs. We have proposed this hemophilia modeling since the first international consensus conference held in Toronto in 2003 and in subsequent consensus documents [18].

In Canada, the Canadian Hemophilia Society (CHS) has worked closely with other blood disorder groups including the HAE patient and physician groups to bring the HAE treatment model in Canada up to the standards of the CHS and the Canadian National Rare Blood Disorders Organizations (NRBDO) meet regularly to share their experiences (last NRBDO meeting was held in Mississauga, Ontario, Canada, November 2009 with proceedings published: http://www.hemophilia.ca/files/NRBDO%202009%20Conference%20Proceedings%20V2.pdf). Hemophilia care in Canada is provided through 26 comprehensive care clinics with treatment products distributed and costs covered under these centres. Blood products in Canada are funded by Provincial Territorial Health Agencies and distributed free to patients through hospital blood banks coordinated through Canadian Blood Services and Hema-Quebec. Despite the hemophilia home care and comprehensive care clinic model being in existence since 1973 [19], progress in the development of comprehensive care clinics and home care for HAE has lagged. In centres where population is not large, HAE clinics sharing with hemophilia clinics would be most practical. Dr. Wolfhart Kreuz and his group in Frankfurt, Germany and Dr. Bruce Ritchie and his group in Edmonton, Alberta, Canada are prime examples of such combined hemophilia/HAE clinics. In a few large population areas, stand-alone HAE comprehensive clinics have developed. Similar to the hemophilia model, and given that health care in Canada falls under the jurisdiction of Provinces and Territories, we have encouraged development and recognition of Provincial and Territorial HAE comprehensive care programs partnering hemophilia and other NRBDO clinics. This would ensure that patients have access to comprehensive care clinics for HAE and that funding for diagnosis therapy and management of HAE models hemophilia care. With the advent of non blood product therapies for HAE, this mainly blood product based hemophilia model may be more difficult to achieve but still is one of the best models for comprehensive HAE care to emulate. Canadian Provincial and Territorial Program global funding for rare disorders like hemophilia, HAE, and immunedeficiency would ensure that patients have access to the safest most cost effective therapy regardless of ability to pay. Who will pay for expensive therapies encountered in rare disorders like hemophilia, HAE, and immunedeficiency remains one of the elephants in the room for discussion.

Similar to hemophilia where therapy was initially given only for bleeding events, HAE therapy started with treatment of angioedema events. In hemophilia, prophylaxis of bleeding events was developed using replacement products and through the Association of Canadian Hemophilia Clinic Directors and their management system and national data collection (Canadian Hemophilia Assessment and Resource Management Systems, CHARMS), the CHS was able to show the cost benefit to hemophilia prophylaxis [20]. In this case, joint damage from bleeding into joints along with death from trauma or spontaneous bleeds could be measured. That is, there was long term tissue damage demonstrable in joints. Unlike hemophilia, if one does not die of the angioedema of the airway, there are usually no long term sequelae to tissues and organs. However, similar to hemophilia there is great impairment of quality of life with recurrent frequent angioedema events with abdominal pain syndromes and temporarily disfiguring peripheral swellings with incapacity for days at a time resulting in significant time away from school, work, social events, family life, and reduced productivity. Similar to hemophilia, it will become critical to have a national data base registry to compare various approaches such as pharmaceutical prophylaxis with androgens such as danazol [16-18,20] versus early angioedema event treatment on demand versus prophylaxis with regular once or twice weekly C1INH replacement infusions. Such registries will also allow early detection of adverse events with newer treatment approaches. The treatment costs for HAE are similar to the annual patient cost for hemophilia and the incidence of severe HAE is similar to severe hemophilia A or B. The HAE community is asking for nothing more than the equivalent care model existing for hemophilia including comprehensive care clinics, program funding, home care, self and assisted treatments. Comprehensive care clinics are needed in the same communities as the hemophilia model (26 comprehensive care centres currently for Hemophilia in Canada). With the many similarities to hemophilia, HAE centres could easily be sprouted from or in collaboration with hemophilia comprehensive care centres.

#### Immunedeficiency

Similar to many patients with immunedeficiency (ID) who are not able to make circulating plasma proteins (usually immunoglobulins) and similar to hemophilia, HAE patients are deficient in plasma proteins that are replaceable by donated human plasma concentrates. Unlike HAE and hemophilia, the blood protein missing in most immunedeficiencies (specific immunoglobulins) will not be easily amenable to development of recombinant products. Similar to HAE and hemophilia, gammaglobulin preparations are expensive but in the case of ID are used only prophylactically to prevent infection rather that to treat acute infection events [21]. Similar to HAE, such blood products for ID patients are given either intravenously (IV) or subcutaneously (SC) with care best accomplished through comprehensive care clinics with central blood product distribution and monitoring through such clinics [21]. Home care and home self or assisted administration of these products can easily be modeled after the hemophilia comprehensive care clinic and home therapy models. Similar to HAE, since many patients are not cared for by hemophilia physicians, development of such comprehensive care clinics and home self or assisted therapy has been slow to develop. Blood products for ID are funded in Canada through Provincial Territorial ministries of health managed through Canadian Blood Services or Hema-Quebec distributing blood products through hospitals into comprehensive care clinics and then into home therapy. Where possible, merging of Hemophilia and HAE clinics with ID clinics would allow resource sharing including home care IV and SC teaching, blood product monitoring including blood-borne pathogen surveillance, and data base management. Where population warrants, stand-alone clinics for each of hemophilia, HAE, ID could develop but in the majority of the 26 hemophilia comprehensive care jurisdictions, combined clinics with HAE and ID would be economical and the most rapid way of introducing home care, home self and assisted infusion programs.

#### Anaphylaxis

Similar to patients with food or stinging insect anaphylaxis who are usually prescribed two adrenaline pens for self or assisted intramuscular administration at times of anaphylactic events, HAE patients are recommended to carry two doses of therapy for IV or SC therapy of acute angioedema events. The cost of an adrenaline pen however is about one tenth the cost of current single administration of HAE treatment. Adrenaline pens are pharmaceuticals on prescription and as such are not covered unless one has drug plan coverage of some sort. Many patients do not have such coverage and despite the possible fatal outcome of an anaphylactic event, they often choose not to carry an adrenaline pen because of cost. Cost and product outdating are significant considerations preventing appropriate product carriage. Ability to pay should not be the factor deciding whether a patient or family carries this life saving adrenaline [22]. Not all anaphylactic events are fatal and most angioedema events in HAE are self-limited. However, one cannot easily predict at the start of an anaphylactic event or airway HAE swelling event whether left untreated this will result in death. This is therapeutic roulette. The playing field between the rich and poor should be leveled in making decisions to carry the adrenaline pen in anaphylaxis patients and carrying various treatment options in HAE and whether to use the life-saving product early in an event. Current blood product therapies for HAE require IV infusion with SC products being investigated. SC administration may be easier to teach and more readily administered. If pharmaceutical non blood product SC agents are licensed, they may not be affordable for a significant portion of the population - either because drug plan coverage is not available for pre-existing illness or because a patient or family may not be able to afford this option without drug plan coverage. The playing field for these HAE life-saving therapies should be leveled by providing all expensive therapeutic products for HAE through Provincial and Territorial HAE programs through comprehensive care clinics whether blood derivative or traditional pharmaceutical agents. Comprehensive care centres can ensure appropriate use and safety monitoring and carry out cost benefit analysis.

#### Asthma

Similar to asthmatics who may experience flares of their wheezing episodes such as around viral infections or stressful times, HAE patients may have flares of their angioedema events around intercurrent illness and particular around stressful events. In asthma, the approach to inhaled steroid with or without long-acting bronchodilator is a step-up approach when flaring and step-down approach when stable. Prophylaxis for angioedema events in HAE may become necessary during stressful times like seeking employment, illness in family members, intercurrent illnesses in the patient, exam times, tight economic times and the like. Patients may need to give more frequent early on demand treatments or move onto prophylaxis one to three or more times weekly. Such HAE prophylaxis to date has been investigated on a continual approach rather than perhaps the step-up, stabilize, step-down approach of asthma. What was surprising in asthma therapy is that the step-up, step-down approach led to better asthma stability in the long run with fewer hospital ER visits and indeed significant less product use compared to regular daily higher dose asthma inhaler use [23]. This was not predicted before being studied and results in improved outcome and considerable cost savings. HAE therapists need to investigate this similar step-up, stabilize, step-down approach through self or assisted preferably home therapy models using large national data base registry data collection. Superiority or non inferiority studies of the two approaches should be done to find the safest most cost effective care model for HAE. One may be surprised as with asthma therapy. However, the goal of therapy is to normalize the lives of patients with HAE and not merely intervene in attacks. Prophylaxis with C1INH now has the potential of nearly eliminating angioedema events.

Annual prophylaxis with danazol 200 mg daily (less than $1000 Canadian per annum) costs less than one therapeutic intervention with the newer therapies for HAE such as with C1-INH plasma or recombinant replacement, bradykinin receptor antagonist, or kallikrein inhibitor (all appear to be significantly greater than $1000 Canadian per treatment). Regular prophylaxis with C1INH is clearly more costly than on demand therapy but similar to hemophilia may achieve near normalization of patient lives. Estimates for annual drug costs utilizing weekly or twice weekly prophylaxis using C1INH range from $100,000 to $200,000 Canadian depending on actual cost per infusion which varies between countries. Economic costs have been reviewed by Wilson et al [24] who estimated average HAE cost of $42,000 US up to $96,000 for more severe patients. Indirect costs added another $16,000 annually. If using routine prophylaxis for patients experiencing one swelling event per month and assuming swelling events are thereby reduced to near zero, the cost of regular versus on demand therapy would be increased from 12 infusions to twice weekly prophylaxis of 104 infusions - an eight to ten fold increase in cost. Assuming $1500 Canadian cost per infusion, this would increase cost from on demand $18,000 Canadian per annum to $156,000 Canadian per patient (assuming no zero break through swelling events). Cost benefit must be closely weighed and different conclusions are likely between countries and health care groups. However, this would reduce patient incapacity from roughly 40 to 50 days per year to near normal life or one or two breakthroughs per year with incapacity of 4 to 8 days. A step-up therapy with flaring, stabilize, and then reduce back to on demand is in use in many clinics in Canada and depending on the patient, is likely the most cost effective model but may still not normalize life as well as regular weekly prophylaxis. This needs careful study and a national program similarly modeled to the Canadian hemophilia program could compare such approaches. Regular prophylactic therapy appears to nearly normalize HAE patient life. Investigating how, when, and in whom to move between danazol prophylaxis to on-demand angioedema treatment to regular short or long term prophylaxis remains to be defined.

### Patient Group Perspective

Similar to the six Hungarian-sponsored HAE Workshops as indicated in their publication [25], it is appropriate that Patient Groups participate in HAE management consensus discussions to share the patient perspective of HAE management and to help reflect on the development of comprehensive care clinics, home therapy programs, and overall management of HAE. Previous international consensus document processes included Patient Group participation in discussion, approval, and co-authoring. Patient groups should participate in and coauthor consensus treatment documents affecting their care [1,18,26]. Patient groups such as hemophilia, HAE, ID should share their experiences and where possible work toward common disease management models and funding with the National Rare Blood Disorders Organization in Canada and their international meetings being one example (last NRBDO meeting was held in Mississauga, Ontario, Canada, November 2009 with proceedings published: http://www.hemophilia.ca/files/NRBDO%202009%20Conference%20Proceedings%20V2.pdf). Patient groups for rare disorders such as hemophilia, HAE, ID should continue to share experiences, resources, and work together for Provincial and Territorial Global Program status to achieve what has worked so well in the hemophilia model: Comprehensive Care Centres across Canada with home care models and self or assisted administration of treatment modalities. Expensive therapies required for these disorders require such Comprehensive Treatment Centre approaches and global funding so that ability to pay does not determine access to the now available life-saving and life-normalizing therapies [20,24].

Proposed Changes Circa December 2010 to the 2010 International Consensus Algorithm for the Diagnosis, Therapy and Management of Hereditary Angioedema published earlier this year in this Journal (Bowen et al. *Allergy, Asthma & Clinical Immunology *2010, 6:24 - http://www.aacijournal.com/content/6/1/24) [1]:

With the publication of several Phase III Clinical trials in HAE prophylaxis and therapy [2-8], some changes to the previously published guidelines are proposed recognizing that new international consensus guidelines will hopefully soon follow through Dr. Cicardi's group.

#### I. HAE Diagnosis Algorithm: See Figure 1 (2010 XII 21)

**Figure 1:**
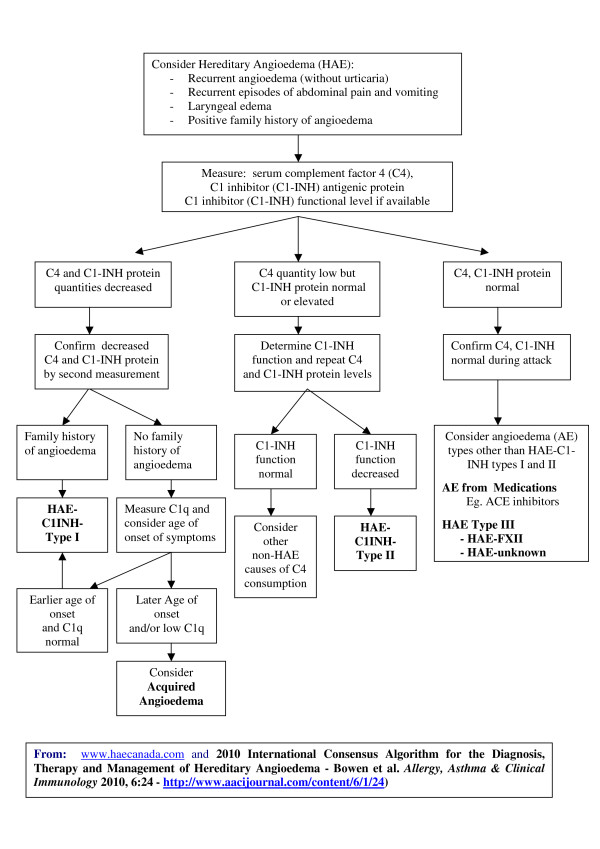
**Hereditary Angioedema - HAE - Diagnostic Algorithm 2010 XII 21**.

No changes are proposed to Figure 1 from the 2010 Consensus document (redated December 21, 2010). For discussion see the HAE Diagnosis Algorithm section in Bowen et al. *Allergy, Asthma & Clinical Immunology *2010, 6:24 - http://www.aacijournal.com/content/6/1/24[1].

#### II/III/IV. Baseline laboratory testing at diagnosis at any age and follow up, Vaccination recommendations and Medications to avoid in patients with HAE

No changes are recommended from the 2010 Consensus document.

See these sections in Bowen et al. *Allergy, Asthma & Clinical Immunology *2010, 6:24 - http://www.aacijournal.com/content/6/1/24[1].

#### V. Short-Term Prophylaxis - see Figure 2 (2010 XII 21)

**Figure 2:**
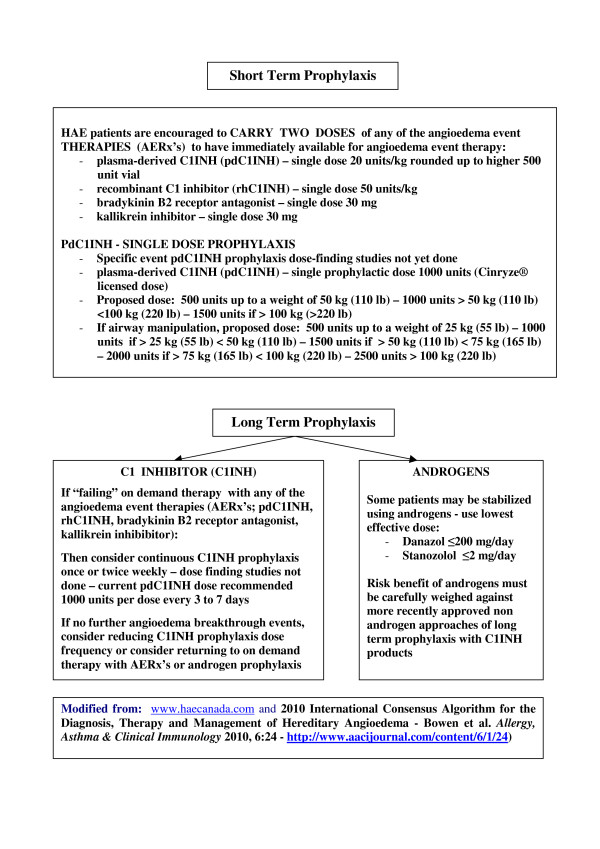
**Hereditary Angioedema - HAE - Prophylaxis Algorithm 2010 XII 21**.

Short term prophylaxis is defined as any prophylaxis intervention intended to protect against an angioedema event with the intent of discontinuing prophylaxis once the indication for prophylaxis has passed. Such indications include medical or dental interventions including endoscopies, dental manipulations, minor or major surgical interventions or stressful events including exam times, job interviews, significant family events, interpersonal relationship upsets and the like. If a particular procedure or personal event is determined to have a low risk of inducing an angioedema event, specific event prophylaxis may be declined but any one of the angioedema event treatments (AERx's; see treatment section below) should be immediately available: plasma-derived C1INH, pdC1INH; recombinant C1INH, rhC1INH; bradykinin B2 receptor antagonist, or kallikrein inhibitor.

HAE patients are encouraged to CARRY TWO DOSES of any of the angioedema event THERAPIES (AERx's) to have immediately available for angioedema event therapy at all times:

- plasma-derived C1INH (pdC1INH) - single dose 20 units/kg rounded up to higher 500 unit vial

- recombinant C1 inhibitor (rhC1INH) - single dose 50 units/kg

- bradykinin B2 receptor antagonist - single dose 30 mg

- kallikrein inhibitor - single dose 30 mg

For specific event angioedema prophylaxis, C1INH replacement therapy is likely the most predictable but this has not specifically been studied for individual event prophylaxis. Prophylaxis trials have been for the general prevention of overall angioedema events and not for specific event prophylaxis where short term prophylaxis is desired. Dose finding for C1INH or other agents in this setting is needed. PdC1INH Cinryze^® ^is FDA approved for angioedema prophylaxis at 1000 units regardless of patient weight http://www.cinryze.com/documents/cinryze-prescribing-information.pdf. PdC1INH Cetor^® ^Sanquin has been registered in the Netherlands since 1997 and lists prophylaxis as well as therapy as indications in its monograph at a fixed dose of 1000 units http://www.sanquin.nl/sanquin-eng/sqn_products_plasma.nsf/8551110e498bd2c8c12572110034decf/11343072be4286d2c125702a004a4e50/$FILE/Cetor%20SPC.pdf. As of October 2010, pdC1INH Berinert^® ^monograph still does not list prophylaxis as an indication http://www.cslbehring.ca/docs/391/332/Berinert_engPM_approved_13Oct2010.pdf. Recombinant rhC1INH Rhucin^® ^Ruconest^® ^is not yet approved for prophylaxis but phase II trials using 50 units once weekly have been announced in news release only November 29, 2010 http://www.biovitrum.com/en/Investors--Media/News/Pharming-Announces-Topline-Study-Results-On-Prophylactic-Use-Of-Ruconest-In-Hereditary-Angioedema/. Pediatric prophylactic dose finding for C1INH has not been done. Cinryze^® ^pediatric and adult prophylaxis studies were recently presented at the World Allergy Organization Meeting in Dubai and used 1000 unit prophylaxis regardless of weight or age 9 [27,28]. I propose using a per weight approach to prophylaxis be studied along the original HAE C1INH dose guidelines: 500 units up to a weight of 50 kg, 110 lb; 1000 units if greater than 50 kg, 110 lb and less than or equal to 100 kg, 220 lb; 1500 units if greater than 100 kg, 220 lb. If airway manipulation considered such as laryngeal intubation, it would seem prudent to prophylax with 20 units/kg rounded up to the whole 500 unit vial (500 units up to and including a weight of 25 kg, 55 lb; 1000 units if greater than 25 kg, 55 lb and less than or equal to 50 kg, 110 lb; 1500 units if greater than 50 kg, 110 lb and less than or equal to 75 kg, 165 lb; 2000 units if greater than 75 kg and less than or equal to 100 kg, 220 lb; 2500 units if greater than 100 kg, 220 lb). A second equal dose to be immediately available for infusion if needed.

If there is a risk that an event may induce angioedema, then pdC1INH prophylaxis may be the most reliable prophylaxis. If no prophylaxis is chosen because of low risk of inducing angioedema, then AERx's (pdC1INH, rhC1INH, kallikrein inhibitor, bradykinin b2 receptor antagonist) should be immediately available and should be used as early in a swelling event as possible. The optimal dose for pdC1INH prophylaxis for procedures has not yet been established. With the availability of pdC1INH, there appears to be little role for androgen, antifibrinolytic, or plasma prophylaxis for events that have a risk of inducing angioedema. Prophylaxis indications with other AERx's await further study. For information regarding androgen, antifibrinolytic, or plasma prophylaxis, see the 2010 Consensus document Bowen et al. *Allergy, Asthma & Clinical Immunology *2010, 6:24 - http://www.aacijournal.com/content/6/1/24[1].

##### V.3. Pregnancy

No changes are recommended from the 2010 Consensus document.

See these sections in Bowen et al. *Allergy, Asthma & Clinical Immunology *2010, 6:24 - http://www.aacijournal.com/content/6/1/24[1].

PdC1INH prophylaxis is the safest prophylactic agent during pregnancy [1,29,30]

##### V.4. Pediatrics

No changes are recommended from the 2010 Consensus document.

See these sections in Bowen et al. *Allergy, Asthma & Clinical Immunology *2010, 6:24 - http://www.aacijournal.com/content/6/1/24[1,31].

#### VI. Long-Term Prophylaxis: See Figure 2 (2010 XII 21)

Consensus recommendations are being prepared by Dr. Marco Cicardi's publication group. It is likely that each country and each health care unit will come to its own conclusions about guidelines for long term prophylaxis. It should be noted that: the number of events per year does not predict severity of the next event nor whether the first or next event will be an airway event. Cost benefit safety analyses will await superiority or non-inferiority studies between various approaches and these will likely be best facilitated by national and international data base registries. Regular prophylaxis with C1INH appears to nearly normalize HAE patient lives with significant reduction in angioedema events but with greatly increased medication costs as outlined above.

##### VI.1. 17-alpha-alkylated anabolic androgens

For discussion of use of anabolic androgens including side effect profile, see this section in the 2010 Consensus document Bowen et al. *Allergy, Asthma & Clinical Immunology *2010, 6:24 - http://www.aacijournal.com/content/6/1/24[1,15-17,32].

If danazol prophylaxis requires greater than 200 mg daily, the risk benefit versus other prophylaxis such as with C1INH should be considered [1,15-17,32]. However, this is not clearly evidence based and patients may be danazol intolerant at lower doses, may not wish to consider androgen therapy (particularly females; pregnancy; prepubertal children), or may tolerate higher doses safely if close monitoring is in place [1,15-17,32]. We await further guidance from Dr. Marco Cicardi's publication group from the September 2010 Italy meeting. With the use of early AERx's on demand or C1INH prophylaxis, some groups are becoming reluctant to exceed 200 mg daily danazol equivalent androgen prophylaxis. The annual cost of 200 mg daily danazol is under $1000 Canadian which is less than a single treatment with any of the AERx's. Cost benefit safety superiority or non inferiorty comparisons including quality of life will be essential for health care groups to recommend and for patient groups to accept therapeutic guidelines. It should not be a requirement for patients to have failed danazol before considering use of other AERx on demand or C1INH prophylaxis approaches. Patient preference should be a major consideration.

##### VI.2. Antifibrinolytic Agents (AFs)

See this section in the 2010 Consensus document Bowen et al. *Allergy, Asthma & Clinical Immunology *2010, 6:24 - http://www.aacijournal.com/content/6/1/24[1].

With the availability of low to moderate dose Danazol prophylaxis or early on demand AERx's or C1INH prophylaxis, many clinicians have abandoned use of AFs outside of the pediatric setting [14,31].

##### VI.3. C1 inhibitor replacement therapy (C1INH)

Home C1INH self-infusion programs should be offered to patients (created similar to hemophilia self-infusion programs which have existed for 35 years [1,19,33-36]. The dose including dose per kg for prophylaxis has not been fully established and appears to need large dose finding studies. PdC1INH therapeutic dose for many years in Europe was a single 500 unit vial [1,18,26,37,38]. With only a small clinical trial with Berinert^®^, the therapeutic Berinert^® ^dose was increased to 20 units per kg. This therapeutic dose needs further study as does the prophylactic dose of C1INH. PdC1INH Cinryze^® ^is licensed at 1000 units but I have not seen the dose finding data versus 500 unit. Dose per kg does not appear to have been studied. PdC1INH Cetor^® ^is licensed at 1000 units therapy or prophylaxis. Deciding how many swelling events per year (such as one swelling event per month) or number of days of disability per year to justify regular prophylaxis is hard to arbitrarily set and perhaps should better be individualized. There may be a progressive approach from early use of AERx's with prodromal symptoms to early on demand administration of AERx's early in a clearly established attack to regular once or twice per week prophylaxis with pdC1INH or once weekly under study for rhC1INH. As outlined in the Asthma Model comparison, step-up, stabilize, step-down approaches to early AERx or C1INH prophylaxis should be studied with careful superiority or non-inferiority designed trials and large date base registry support.

###### VI.3.a Plasma-derived C1 inhibitor - pdC1INH

**Cinryze^® ^**from ViroPharma is FDA approved for adolescent and adult prophylaxis at a dose of 1000 units intravenously every three or four days (see FDA approved package insert:

http://www.fda.gov/BiologicsBloodVaccines/BloodBloodProducts/ApprovedProducts/LicensedProductsBLAs/FractionatedPlasmaProducts/ucm150480.htm) [2,39]. Prophylaxis with pdC1INH is not 100% effective http://www.cinryze.com/documents/cinryze-prescribing-information.pdf[2]. Reports of regular prophylaxis (1000 units every 3 to 7 days) in pediatric and adult patients have been reported at the World Allergy Organization International Scientific Conference in Dubai December 7^th^, 2010 [27,28]. Adult median attack rates reduced from a 3 per month to 0.2 per month with 35% of patients reporting no attacks. Pediatric attack rates reduced from 4.4 per month to 0.4 to 0.7 attacks per month. Again dose finding for dose per kg and frequency of administration are not fully established.

**Cetor^® ^**from Sanquin is licensed in the Netherlands for therapy or prophylaxis at a dose of 1000 units intravenously with no frequency recommendation http://www.sanquinreagents.com/sanquin-eng/sqn_products_plasma.nsf/8551110e498bd2c8c12572110034decf/11343072be4286d2c125702a004a4e50/$FILE/Cetor%20SPC.pdf.

**Berinert^® ^**from CSL Behring is approved for therapy in many countries around the world including Europe and by USA FDA (see FDA approved package insert:

http://www.fda.gov/BiologicsBloodVaccines/BloodBloodProducts/ApprovedProducts/LicensedProductsBLAs/FractionatedPlasmaProducts/ucm186264.htm) but **not yet licensed for prophylaxis in North America**. Berinert^® ^has been used for many years in Europe however early in attacks and in Europe [40,41] and in Canada on Special Access for prophylaxis or early intervention. **We await publication of prophylaxis data from CSL Behring**.

###### VI.3.b Recombinant C1-INH

Conestat alfa, Rhucin^® ^in non-European countries, Ruconest^® ^in Europe, Pharming, is recombinant human C1-INH produced in transgenic rabbit milk is approved for treatment of HAE by the European Medicines Agency's (EMA) Committee for Medicinal Products for Human Use (CHMP) and is under FDA review. **Not yet licensed for prophylaxis **but preliminary Phase II study has been announced by Pharming at 50 U/kg weekly with baseline attack rates of 0.6 attacks per week being reduced to 0.25 attacks per week 9 http://www.biovitrum.com/en/Investors--Media/News/Pharming-Announces-Topline-Study-Results-On-Prophylactic-Use-Of-Ruconest-In-Hereditary-Angioedema/. **We await publication of prophylaxis data from Pharming**.

#### VII. Treatment of Acute HAE Attacks - see Table 1 (2010 XII 21)

**Table 1:** Treatment of Acute Hereditary Angioedema - HAE - Attacks - 2010 XII 21

ANGIOEDEMA EVENT THERAPIES (AERx's)	
**TREAT AS EARLY AS POSSIBLE IN AN ATTACK**	

**Plasma-derived C1 INH (pdC1INH) (intravenous)**	○ **Berinert^® ^CSL Behring - approved in many countries (including Europe and North America)**
	▪ **20 units/kg intravenously (FDA licensed dose)**
	○ **Cetor^® ^Sanquin - approved in the Netherlands 1997**
	▪ **1000 units intravenously**
	○ **Cinryze^® ^ViroPharma - under review for therapy**

**Recombinant C1INH (rhC1INH) (intravenous)**	○ **conestat alfa, Rhucin^® ^non-European and Ruconest^® ^in Europe; Pharming**
	▪ **50 units/kg intravenously**
	○ **approved for use by the European Medicines Agency (EMA) for use in the European Union 2010**
	○ **under review in North America**

**Bradykinin B2 receptor antagonist (subcutaneous)**	○ **Icatibant - 30 mg (subcutaneous) (Firazyr^® ^(Jerini/Shire)**
	○ **approved for use by the European Medicines Agency (EMA) for use in the European Union 2008**
	○ **not yet approved in North America**
	
**Kallikrein receptor antagonist (subcutaneous)**	○ **Ecallantide, Dyax, DX-88, Kalbitor®**
	○ **30 mg subcutaneously**
	○ **approved USA 2009 - not yet available in Canada**

*Modified from: www.haecanada.com and **2010 International Consensus Algorithm for the Diagnosis, Therapy and Management of Hereditary Angioedema - Bowen et al. *Allergy, Asthma & Clinical Immunology *2010, **6**:24 - http://www.aacijournal.com/content/6/1/24)**

##### We recommend treating attacks as early as possible

Evidence based consensus was discussed at the meeting organized by Dr. Marco Cicardi, Italy, September 2010 with publications in preparation by his groups. Evidence based approaches for treatment will appear there. There are now published phase III clinical data for pdC1INH, rhC1INH, icatibant, and ecallantide providing level one evidence for use of these products for therapy of various angioedema events in HAE. No superiority nor lack of inferiority head-to-head trials between the four licensed therapies have been conducted to date. Although not specifically studied, no angioedema attack site or type has been shown to require more or less therapy than others.

###### VII.1. Plasma-derived C1-INH - PdC1INH

PdC1INH has been the first line therapy for several decades around the world particularly in Europe (more than 25 years experience in Europe with pasteurized Berinert) [1,18,26,37,38,41,42]. **Berinert^® ^**from CSL Behring was licensed by USA FDA October 9^th^, 2009 for therapy of HAE events and licensed in many other countries for many years and Phase III clinical trial Level one evidence is published [6,7]. **Cetor^® ^**from Sanquin has been available in The Netherlands since 1997. Berinert^®^, CSL Behring, has been shown to be more effective than placebo for therapy of acute angioedema attacks at a dose of 20 units/kg (see package insert reference above) [7]. However, use in the past has been 500 to 1500 units [1,18,26,37,38,41,42]. Cetor dose recommendation is 1000 units - http://www.sanquin.nl/sanquin-eng/sqn_products_plasma.nsf/8551110e498bd2c8c12572110034decf/11343072be4286d2c125702a004a4e50/$FILE/Cetor%20SPC.pdf. Cinryze^® ^treatment clinical trials are currently being evaluated by regulatory groups and I believe used a similar 1000 unit infusion approach). PdC1INH has been well tolerated and pathogen transmission attributed to new generation pdC1INH is very rare [37,38]. As pdC1INH is a blood product, annual recipient hemovigilance and vein-to-vein tracking are essential (tracking and hemovigilance similar to home therapy programs for Hemophilia Comprehensive Clinics). The dose of pdC1INH was traditionally 500 unit single infusion and second infusion was rarely needed [1,18,26,37,38,42]. Against this large successful clinical experience, the relatively small Berinert^® ^pivotal licensing studies showed 10 unit per kg no better than placebo whereas the 20 unit per kg was shown effective against placebo. Safety efficacy of doses of 20 units per kg were studied but not doses rounded off to the next highest 500 unit vial (no particular reason to expect increased toxicity but not specifically studied in these protocols). The dose recommended in the first consensus conference of 500 units for < 50 kg; 1000 units for 50 kg or greater up to <100 kg; and 1500 units for > 100 kg has not been formally studied. It would seem prudent for health groups to consider studying this dose approach in superiorty or non-inferiority studies as the cost saving would be significant and the safety of rounding off to the next highest vial dose for at least 20 units per kg could similarly be studied. Although pdC1INH is not yet approved for pediatric use, it has been in wide clinical use in pediatric patients in Europe for years [31]. Although pdC1INH is not yet approved for use in pregnancy, it has been in wide clinical use in pregnancy and lactation in Europe for years [29,30]. No AERx is specifically approved for pediatric use, in pregnancy, nor in lactation.

###### VII.2. Bradykinin B2 receptor blocker, Icatibant

Icatibant (Firazyr^® ^from Jerini/Shire) is a small peptide, bradykinin B2 receptor blocker approved for use in treatment of HAE in the European Union. Dose is 30 mg subcutaneously in adults. Pediatric experience is pending. Although not usually needed, the dose can be repeated six hourly twice more if needed (see package insert for Firazyr^®^). Local reactions are common with injection. Phase III clinical trial Level one evidence is published [4].

###### VII.3. Kallikrein inhibitor, Ecallantide

Ecallantide, DX-88, Dyax, Kalbitor^® ^is a small peptide, kallikrein inhibitor approved for treatment of HAE in the USA since December 2009. Dose is 30 mg subcutaneously (adults). It is not recommended for self infusion at this time because of a small risk of anaphylaxis and is being further studied in phase IV clinical trial. Phase III clinical trial Level one evidence is published [5].

###### VII.4 Recombinant C1-INH, rhC1INH

Conestat alfa, Rhucin^®^, Ruconest^® ^is recombinant human C1-INH produced in transgenic rabbit milk (4,23) is approved for treatment of HAE by the European Medicines Agency's (EMA) Committee for Medicinal Products for Human Use (CHMP) and is under FDA review.

Phase III clinical trial Level one evidence is published and showed 100 units per kg no better than 50 units per kg [3].

#### VIII. Comprehensive Care Clinics - Home Therapy: see Table 2 (2010 XII 21)

**Table 2:** Comprehensive Care Clinics for Hereditary Angioedema - 2010 XII 21

Comprehensive HAE Clinics will Provide:	
1. Best Clinical Treatment outcomes including:	a. a comprehensive care team made up of nurse coordinator, clinician, social worker, data manager, pain management specialist, genetic counselor, and administrative support; b. access to specialized diagnostic testing; c. access to home treatment; d. a networked Patient Information System to facilitate product recalls - collect data on therapy outcome measures and safety, and facilitate participation in clinical trials e. access to clinical advances as they become available; f. access to 24 hour support; g. access to up-to-date standards of care, including standardized wallet cards; h. tracking and intermittent audit of quality outcomes including beneficial and adverse outcomes through secure, comprehensive and networked data management.

2. Education of patients and staff regarding:	a. responsible Self/Family Care (home care model) with home and self infusion/administration instruction and support; b. developments in the cause, diagnosis, treatment, outcomes, and prognosis of HAE c. changes in the administrative management of the clinic

3. An environment conducive to research including:	a. access to and support for clinical trials of new treatments; b. access to and support for translational research in diagnosis and prognosis; c. access to and support for psychosocial research such as quality of life studies.

4. An advisory or oversight board with patient group representation for each clinic	

**(Modified by permission from: **www.haecanada.com - comprehensive care clinics)

Comprehensive Patient Care Clinics: Clinical care, Education, and Research

Comprehensive care for HAE is based on the recognition that HAE is a chronic disease and care is complex, requiring a highly specialized and multidisciplinary approach. A comprehensive care clinic must provide accountability for in-hospital and home use of expensive and potentially toxic treatments, track outcomes (both beneficial and adverse), and develop and meet Standards of Care for HAE.

Comprehensive care clinics for immunedeficiencies, rare blood disorders, hemophilia, cystic fibrosis, asthma, cancers and many other disorders have improved survival [43,44] and contributed to improved standard of care for these disorders (see proceedings of the Canadian National Rare Blood Disorders meeting:

http://www.hemophilia.ca/en/about-the-chs/collaboration/network-of-rare-blood-disorder-organizations/2009-progress-in-comprehensive-care-for-rare-blood-disorders-conference----presented-by-csl-behring/#c969). Comprehensive care for HAE is based on the recognition that HAE is a chronic disease and care is complex, requiring a highly specialized and multidisciplinary approach. A comprehensive care clinic must provide accountability for in-hospital and home use of expensive and potentially toxic treatments, track outcomes (both beneficial and adverse), and develop and meet Standards of Care for HAE. It is recommended that HAE patients be linked with comprehensive care clinic programs (bringing together clinical care, education and research) to facilitate diagnosis, therapy, management; facilitate data base registries; allow rigorous safety efficacy monitoring of emerging therapies of HAE; and to facilitate access to home therapy programs (similar to the model for comprehensive care of hemophilia) (see blood disorder conference link above) [1,18,19,26,33-36,40]. One clinic model can be found in Table 2[1]. Patients are encouraged to carry "alert" identification (wallet card example may be found at: http://www.haecanada.com/files/WalletCard_Bilingual.pdf) and an accompanying letter indicating the diagnosis of HAE (with type), materials necessary to be carried for care for presentation at airline and other security areas, and outlining instructions for administration of intervention therapy (such as infusion of pdC1INH, rhC1INH, bradykinin B2 receptor antagonist, or kallikrein inhibitor). It is recommended that patients carry two doses of whichever AERx with them and be educated in their administration. It is recommended that HAE organization websites provide infusion instructions for downloading by patients and comprehensive care clinics (example of home infusion technique may be viewed at: http://haecanada.com/infusion/index.html.) Home therapy and particularly home infusion programs should be offered to patients. Such programs should be created similar to hemophilia home infusion programs which have existed for 35 years (see blood disorders link above) [1,18,19,26,33-36,40]. Home care was discussed at the 6^th ^International HAE Conference held in Budapest in June 2009 http://www.haenet.hu/new/program_C1INH2009.pdf and the resulting home care consensus approach has been presented [33]. Home care and self or assisted infusion programs were discussed at Dr. Cicardi's September 2010 meeting with consensus publication of this pending.

#### IX. Pediatrics

Angioedema therapies have been licensed for adults with no pediatric licensing [1,31].

#### X. Pregnancy and Lactation

Angioedema therapies have been licensed for non pregnant, non lactating adolescents or adults and have not been licensed for use in pregnancy nor lactation. There is anecdotal use of pdC1INH use in pregnancy and lactation [29,30].

## Conclusion

Since our first Canadian International Consensus meeting in 2003 [18] when plasma-derived C1-inhibitor concentrates had been available for decades in Europe but not widely outside Europe, many new therapies have emerged in HAE management. Phase III clinical trials have now been reported on and are now licensed in various countries for prophylaxis and therapy of HAE and hopefully together with home care approaches are reducing the morbidity and mortality in this disorder and allowing HAE patients to lead near normal lives. We must strive to elevate the standard of care for HAE patients through comprehensive care clinics and home care programs and institute safety, efficacy, and cost benefit monitoring.

It is important to conduct rigorous phase IV clinical trials preferably utilizing national and international data base registries so that long term safety efficacy data on these therapies can be closely monitored and to allow comparison of cost benefit studies including quality of life issues between the various therapies. Various approaches including early treatment on demand versus step-up step-down versus regular prophylaxis need close study and analyses. This will provide funding organizations and patients better information on which to base their choices of products provided under pharmaceutical plans and the most cost effective product for patient choice. It is exciting to finally have licensed therapeutic and prophylactic medications for treatment of this disorder.

Consensus approaches are only interim guides to chronic and rare diseases such as HAE and should be replaced as soon as possible with more phase III studies, meta analyses, large phase IV post-marketing trials, and head-to-head superiority or non-inferiority studies using data base registry validation of approaches including quality of life and cost-benefit analyses. It is likely that individual countries and health care agencies will create their own guidelines and standards for HAE disease management. Some of these will vary by ability of countries to fund various treatment algorithms, comprehensive care clinics, and the various philosophies of payment systems for health care delivery. Certainly Canadian Provincial and Territorial approaches are in flux. I would urge creation of Provincial and Territorial Program recognition for HAE along with Hemophilia and immunedeficiency and would urge therapeutic agents be funded under and directed through comprehensive care clinics in a network similar to the Hemophilia Comprehensive Care model. This levels the playing field for HAE patients such that any HAE patient can expect the same health care support regardless of ability to pay. The only HAE therapy currently licensed for use in Canada is Berinert^®^. We hope some time in the near future to have the same therapeutic options available in Canada as are available in Europe, the United States, and other parts of the world.

## Competing interests

Dr. Bowen has in the past either entered consultancy with or has been involved in educational programs and their organization, had direct funding from, has been speaker for, or has had consultation agreements with CSL Behring, Dyax, Jerini, Pharming, ViroPharma, Shire. In the past year, Dr. Bowen has sat on a North American Advisory Panel for Shire (November 2010). Dr. Bowen is co-chief editor of Allergy, Asthma and Clinical Immunology.

## Authors' contributions

TB prepared the manuscript.
